# Drug-induced linear IgA bullous dermatosis with extensive mucosal involvement

**DOI:** 10.1016/j.jdcr.2024.07.021

**Published:** 2024-08-10

**Authors:** Tory Starzyk, Jacqueline Nikakis, Risa Ross

**Affiliations:** a Department of Dermatology, HCA Healthcare/USF Morsani College of Medicine GME-HCA Florida Largo Hospital, Largo, Florida; b New York Institute of Technology College of Osteopathic Medicine, Old Westbury, New York

**Keywords:** drug-induced, genital, linear IgA bullous dermatosis, mucosal

## Introduction

Linear IgA bullous dermatosis (LABD) is an autoimmune disease characterized by linear IgA deposition along the skin’s basement membrane, leading to dermoepidermal junction disruption and blister formation.^1^ The association between drugs such as vancomycin (most commonly implicated), nonsteroidal anti-inflammatory drugs, penicillins, cephalosporins, diuretics, anticonvulsants, and LABD is well-established. Although cases with oral involvement and 1 case of idiopathic LABD with localized penile involvement have been reported, cases with extensive mucosal lesions including both oral and genital have not yet been published.^2^^,^^3^ To our knowledge, this case report details the first-reported occurrence of drug-induced LABD with extensive mucosal involvement that includes the penis, oral, nasal, and ocular mucosa.

## Case report

A 45-year-old man with alcohol-related cirrhosis was admitted to the hospital for hepatorenal syndrome and sepsis. He received 1 dose of intravenous (IV) vancomycin at admission and was changed to IV meropenem and IV micafungin 2 days later. Methicillin-resistant *Staphylococcus aureus* nares swab and urine, blood, and abdominal fluid cultures were negative. After 5 days, dermatology was consulted for extensive blistering.

**Fig 1:**
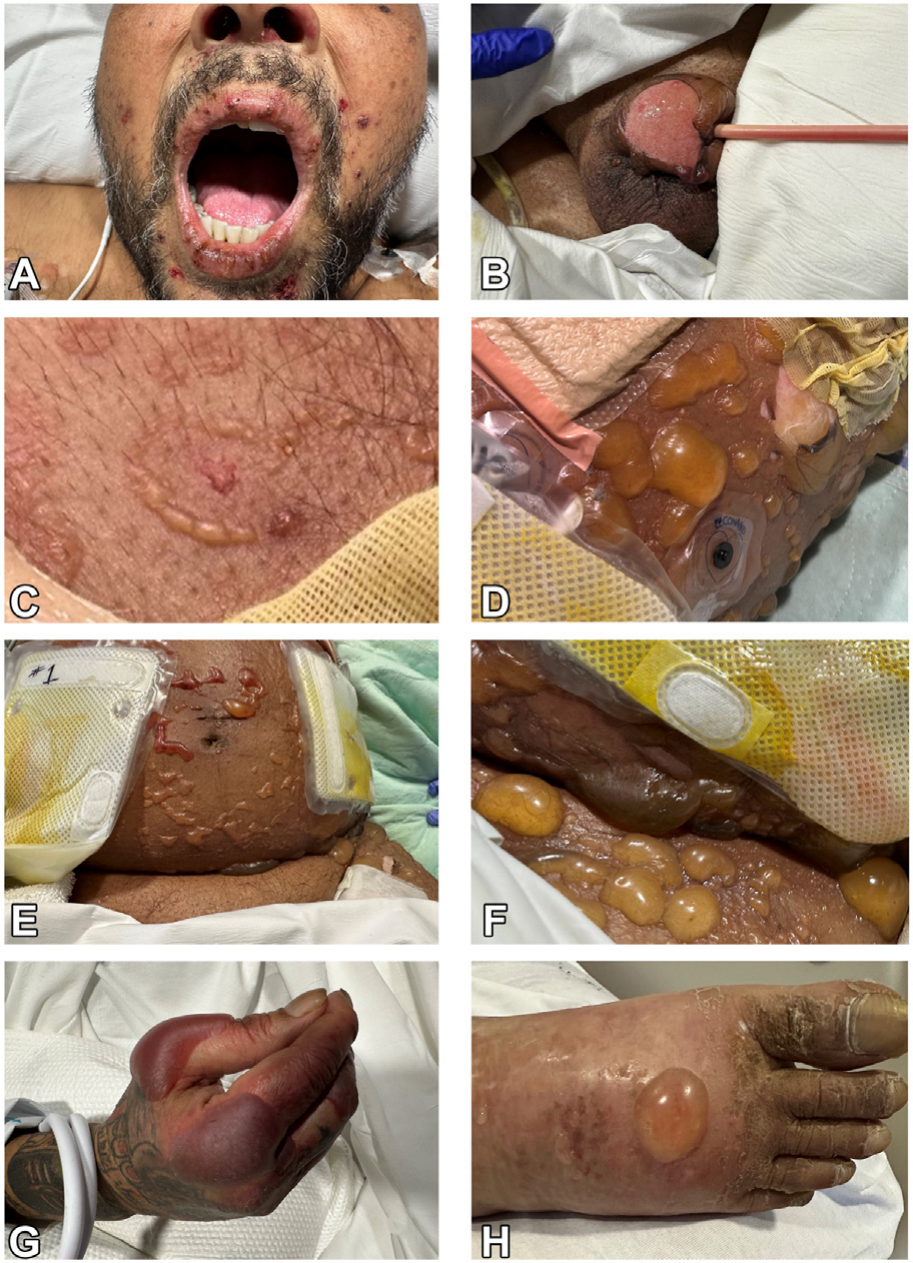
**A,** Hemorrhagic crusted erosions on the lips and nares. **B,** Ruptured bulla on the penis. **C,** “Crown of jewels” vesicles on the chest. **D-F,** Tense vesicles and bullae with erosions on the trunk and groin. **G,** Tense bullae on the hand. **H,** Tense bulla on edematous foot.

Physical examination revealed multiple tense vesicles and bullae on the trunk, arms, axilla, groin, dorsal aspect of the hands, feet, and penis (Fig 1). A single bulla on the trunk exhibited an annular “crown of jewels” appearance. Hemorrhagic crusting on the lips and nares was observed and, despite the absence of ocular lesions, the patient reported conjunctival pruritus. The differential diagnosis included Steven-Johnsons syndrome, edema bullae, pemphigus vulgaris, mycoplasma-induced rash and mucositis, bullous pemphigoid, LABD, and staphylococcal-scalded skin syndrome. The initial plan included topical triamcinolone and mupirocin, electrolyte management, chest X-ray, and 2 punch biopsies on the upper portion of the right arm for hematoxylin and eosin staining and direct immunofluorescence.

**Fig 2:**
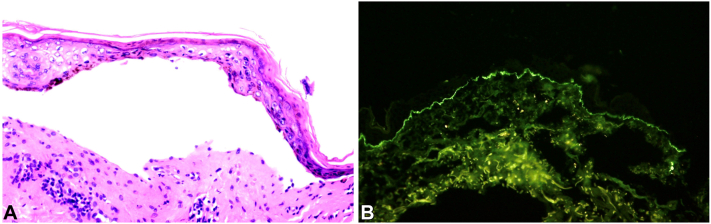
**A,** Hematoxylin and eosin staining—pauci-inflammatory subepidermal blister. **B,** Direct immunofluorescence—linear IgA basement membrane zone deposition. (Original magnifications: **A,** ×40; **B,** ×40).

Chest X-ray was negative making mycoplasma-induced rash and mucositis less likely. Hematoxylin and eosin staining revealed cell-poor subepidermal vesicular dermatitis without confluent epidermal necrosis, which ruled out Steven-Johnsons syndrome (Fig 2). Direct immunofluorescence demonstrated IgA linear basement membrane deposition and was negative for IgG, immunoglobulin M, C3, and fibrinogen. This clinicopathologic presentation supported a diagnosis of LABD. Three days later, although no new bullae were present, existing ones had ruptured, exposing 33% body surface area and warranting transfer to burn unit level of care. The final plan included wound care, electrolyte management, topical triamcinolone for bullae, and topical mupirocin for superimposed impetigo on his lips.

## Discussion

This case highlights a rare occurrence of drug-induced LABD with oral, ocular, nasal, and genital manifestations. Although multiple IV medications were prescribed, the most likely culprit that triggered the LABD in our patient was the single dose of IV vancomycin administered at admission. Onodera et al^4^ reported that between 1981 and 2019, 100 cases of LABD were published with vancomycin being the most common trigger. As the patient only received 1 dose of IV vancomycin, discontinuation of the offending drug did not apply to this case.

Vancomycin is primarily eliminated by the kidneys and 80% to 90% of vancomycin is excreted within 24 hours.^5^ Vancomycin clearance decreases linearly with creatinine. Owing to our patient’s impaired renal function secondary to hepatorenal syndrome, we hypothesize that decreased clearance of IV vancomycin may have exacerbated his condition.

The extent of mucosal involvement in this case, encompassing multiple mucosal surfaces, including the genital area, is exceptionally rare in LABD. Although previous cases of LABD with oral and nasal involvement have been documented, reports of genital involvement are limited, with only 1 prior case described in the literature.^3^ However, this previous case involved localized lesions confined to the penis without concurrent cutaneous manifestations. Additionally, this case was believed to be due to viral or autoimmune LABD in contrast to our case, which we hypothesize is drug-induced. To our knowledge, our case is the first case of drug-induced LABD reported with diffuse blistering encompassing the oral, nasal, ocular, and genital areas.

The management of drug-induced LABD primarily involves discontinuation of the offending medication and symptomatic treatment of skin lesions. In severe cases with extensive blistering, as seen in this patient, supportive measures such as wound care and topical corticosteroids may be necessary to promote healing and prevent secondary infection.

Though infrequent, considering drug-induced LABD as a potential cause for diffuse blistering, encompassing the oral, nasal, ocular, and genital areas is crucial. A multidisciplinary approach and heightened awareness are essential for better managing such cases.

## Conflicts of interest

None disclosed.
